# Evaluation of the Utility of histopathologic exam as a routine in Tonsillectomies

**DOI:** 10.1016/S1808-8694(15)30064-1

**Published:** 2015-10-19

**Authors:** Felippe Felix, Geraldo Augusto Gomes, Bruno Peer de Souza, Gustavo Azeredo Cardoso, Shiro Tomita

**Affiliations:** a 3rd year resident of Otorhinolaryngology - Clementino Fraga Filho University Hospital - Federal University of Rio de Janeiro.; b MD, MS student - Otorhinolaryngology - Clementino Fraga Filho University Hospital - Federal University of Rio de Janeiro.; c Medical Student - Federal University of Rio de Janeiro.; d Medical Student - Federal University of Rio de Janeiro.; e Full Professor and Head of Otorhinolaryngology - Clementino Fraga Filho University Hospital - Federal University of Rio de Janeiro.

**Keywords:** tonsillectomy, histopathologic, cost

## Abstract

Tonsillectomy is one of the most commonly performed procedures of the head and neck. It is performed for a wide variety of indications in both adults and children. It is common to send the material achieved in the surgery to routine histopathologic exam, as to analyze suspected material or for a medical-legal documentation. **Objective:** Analyze the utility and cost of routine histopathologic diagnosis for tonsillectomy. **Methodology:** retrospective study of the histopathologic result of all tonsillectomies between 1978 and 2004 in a university hospital and analyzed the files of the patients with cancer. **Results:** 2103 results of histopathologic exams were analyzed. Of these, only four cases presented any case of malignancy, being all of these non-Hodgkin lymphoma and already suspected before the surgery. **Discussion:** The world literature has encountered similar results and each time more the histopathologic analysis of all cases is questioned. The cost of the exam is high and your results, in the case of malignancy were already knew before the surgery. **Conclusion:** Histopathologic analysis of all tonsillectomies is not indicated. The risks factors established by Beaty should guide the solicitation of the exam, to try to low the costs with unnecessary exams.

## INTRODUCTION

Tonsillectomy is one of the most commonly performed surgeries in the world. It bears a variety of indications; both in adults and children, and the most common indications are upper airway obstruction and recurrent tonsillitis.

It is common to send the specimen for histopathology exam, most of the times as standard procedure in many institutions, either to analyze suspicious material or for legal purposes as proof of its removal^1^. Besides, hidden malignancy, in other words, the finding of malignant neoplasia by chance in a surgical specimen without clinical suspicion before surgery, is an important factor that favors the fact that all physicians should send their surgical specimens to analysis.

This debate about sending or not this material for exam is nothing recent. Starry^2^ and Yarington^3^, in 1939 and 1967, respectively, instructed physicians to request histopathology exam of all specimens. Weibel^4^, in 1965, was the first to speak against the microscopic exam in routine tonsillectomies.

In this study, we retrospectively analyzed 2,103 charts from patients who underwent tonsillectomies regardless of surgical indication, gender and age, aiming at evaluating if the routine histopathology exam is indicated.

## METHODS

We reviewed the files from the Department of Pathology of the University Hospital Clementino Fraga Filho, and collected the histopathology analysis report from palatine tonsils of adult and pediatric patients operated in the last 25 years, from 1978 to 2004, regardless of patient age and surgical indication. Within the group of non-malignant pathologies we included the following cases: follicular hyperplasia, lymphoid hyperplasia, acute and chronic inflammation.

We excluded the biopsy cases in which the entire tonsil was not removed.

Patients that had malignancy in their exam had their charts pulled out and reviewed. Besides, there was a cost analysis for both the hospital and the public health system, and we consulted the literature from the last 20 years on the subject.

The cost of each histopathology exam for palatine tonsils is of about R$ 13.89 per specimen, according to data supplied by the Hospital Financial Department and the rates from the Public Health System. Since in all the cases the tonsillectomy was bilateral, the cost ended up being of R$ 27.78. Data from the private sector vary according to insurance company and the type of insurance plan the patient had subscribed to, and they were not used in this study.

## RESULTS

A total of 2,103 histopathology analysis results were reviewed. Of this sample, only 4 cases presented some type of malignancy, and all of them were non-Hodgkin lymphomas, corresponding to 0.19% of the total. The four cases are reviewed on Table 1.

**Table 1:** Evaluation of malignant cases related to Beaty risk factors.

	Case 1	Case 2	Case 3	Case 4
Age of diagnosis	80	44	28	75
Neck lymphadenomegaly	X	X	X	-
Palatine tonsil asymmetry	X	X	X	X
Mucosal irregularities/alterations felt in the palpation	7	X	X	X
Body symptoms or unexplainable weight losing	X	X	-	-
Past history of immune depression or malignancy	7	7	-	-
Suspicion before surgery	X	X	X	X

X: present; -: absent; ?: undetermined or unavailable

15 cases of squamous cell carcinoma were found in palatine tonsils. These cases were not included in this paper because they were found by lesion biopsy only, and not tonsillectomy, as all the other ones which were evaluated.

## DISCUSSION

The pathological analysis of the surgical material serves to guide patient care and treatment. It also it helps guarantee for the health insurance plans that documented procedures are performed, for legal reasons as well as an educational tool, used to confirm presumed diagnosis. Recently, studies based on the histology of palatine tonsils have shown that in routine cases this exam may not be necessary^5^. These authors show that there is very little chance for a significant pathological finding in the specimens removed by routine indications. Unfortunately this risk is not zero, yet, that is why the need for routine histopathology is still controversial.

In 1996, Dohar and Bonilla^6^, sent a survey to pediatric otolaryngologists in the United States. In this survey they found that 56% of the physicians sent all the removed material to microscopic analysis, 44% only sent them to macroscopic analysis or did not do it at all. Besides, these same authors reviewed 2,000 pediatric tonsillectomies from an American institution without finding significant pathology in cases not suspected from having malignancies before surgery, and its incidence was extremely rare in all the surveys.

Another study from Strong et al.^7^, from 1999, represents a statistically significant change in routine histopathology analysis for tonsillectomies, comparing similar questionnaires applied in 1989 and 1999. About 33% of the interviewees had changed their behavior of sending each and every palatine tonsil to histopathology in these ten years. This trend is uniform both for adults and children, and the major factors mentioned by the interviewees were cost and new information in the otorhinolaryngology literature.

In 1998, after analyzing 476 tonsillectomies, Beaty^8^ proposed risk factors for tonsillar malignancy, which were:

- Previous history of head and neck cancer
- Tonsillar asymmetry
- Visible lesion or hard to the touch when of tonsil palpation
- Unexplained weight loss or constitutional symptoms
- Neck mass

In 1987, Ridgeway et al.^9^, found 5 pediatric patients with non-Hodgkin lymphoma in a 13 year analysis of routine tonsillectomies and recommended the histopathological analysis of all cases in order to try and find nonsuspected malignancies. Notwithstanding, in 2001, Younis et al.^10^, revised these 5 cases and found one or more of the Beaty malignancy criteria in these patients that should have aroused clinical suspicion on the preoperative.

Younis et al.^10^ reviewed 2,438 cases; 2,099 pediatric and 339 adults, and did not find alterations in the pediatric cases and found 40 malignancies in the adult group, 6 lymphomas and 34 squamous cell carcinomas, and all these cases had already been suspected in the preoperative due to the patient’s history and clinical findings.

In 1980, in one of the largest reviews ever carried out, Daneshbod et al.^11^, evaluated 15,120 histopathology analysis of adult and children palatine tonsils and did not find any incidental finding. All the malignancy cases in the study presented some preoperative suspicion, either by clinical history or physical exam.

In our study we found 2,103 histopathology exams for tonsillectomies in a 25 year analysis. There was malignancy in 4 cases only; these patients already presented preoperative clinical suspicion and they all presented two or more Beaty risk factors for malignancy investigation. They were all adults, no malignancy cases were found among the children.

The estimated cost for each conventional pathology exam for the government is currently of R$13.89 per specimen; since for tonsillectomies there are two of them, the cost is twice that price. Younis et al. reported that the cost for the American public sector is US$4.82 for macroscopy and US$12.85 for microscopic analysis. Sanchez et al.^13^ say that in Mexico the cost is US$10.00 for the public health system for a complete pathological analysis and US$50.00 for the private sector. There were no cost data for macroscopic exam separate from the microscopic exam in Brazil and Mexico.

## FINAL COMMENTS

Aware of the low malignancy incidence in palatine tonsils (about 0.19% in our sample), and the possibility of having a preoperative triage through the collection of a thorough history and proper physical exam, it is not worth to spend thousands of Dollars per year by institution in order to obtain routine histopathological exams for tonsillectomies.

Beaty risk factors should guide the request for histopathology, so that we may reduce the cost incurred by unnecessary exams.

As a suggestion for legal proof and proof for the insurance companies, we may use the macroscopic exam, which reduces up to three fold the total cost of the exam.
